# Concanavalin A Protocol for Felid Lymphocyte Culture and Chromosome Preparation

**DOI:** 10.3390/ijms27156662

**Published:** 2026-07-26

**Authors:** Príncia Grejo Setti, Alan Moura de Oliveira, Stéphanie Ferguson Motheo, Otávio Guilherme Gonçalves de Almeida, Thomas Liehr, Marcelo de Bello Cioffi

**Affiliations:** 1 Department of Genetics and Evolution, Federal University of São Carlos, São Carlos 13565-905, SP, Brazil; psetti@estudante.ufscar.br (P.G.S.); alanoliveira@estudante.ufscar.br (A.M.d.O.); 2 Central Paulista University Center, São Carlos 13563-470, SP, Brazil; stephanifm@yahoo.com.br; 3 Wildlife Screening and Rehabilitation Center (CETRAS), Morro de São Bento, Ribeirão Preto 14091-902, SP, Brazil; ogalmeida@rp.ribeiraopreto.sp.gov.br; 4 Institute of Human Genetics, Jena University Hospital, Friedrich Schiller University, 07747 Jena, Germany

**Keywords:** cytogenetics, Felidae conservation, mitogen stimulation, peripheral blood culture, wildlife genetics

## Abstract

Chromosome preparation from peripheral blood in felids is often difficult because lymphocytes respond inconsistently to standard mitogens like phytohemagglutinin (PHA), resulting in low and variable mitotic indices. Here, we present a standardized and reproducible protocol for lymphocyte culture and metaphase chromosome preparation in felids using concanavalin A (ConA) as a mitogenic stimulus. A total of 300 µL of peripheral blood consistently produced optimal chromosome preparations and was cultured in RPMI 1640 medium supplemented with fetal bovine serum, antibiotics, and Concanavalin A (40 µg/mL), then incubated at 37 °C for 70 h. Mitotic arrest was induced with colchicine, and cells were subjected to hypotonic treatment and fixation using methanol–acetic acid before slide preparation and Giemsa staining. This approach consistently yielded high-quality metaphase spreads across the eight felid species examined, with mean values ranging from 32 ± 5 to 55 ± 7 metaphases per slide, while maintaining chromosome morphology appropriate for cytogenetic investigation. Compared with fibroblast-based approaches, this methodology is less invasive and more feasible for clinical and conservation applications because it relies on peripheral blood collection rather than tissue biopsy, reducing the invasiveness of sampling procedures. This optimized lymphocyte culture workflow enhances mitotic yield and repeatability, serving as a significant resource for cytogenetic research, particularly in veterinary genetics, evolutionary biology, and the conservation of endangered felid species.

## 1. Introduction

Cytogenetics is a fundamental tool for studying the eukaryotic genome. Through chromosomal analysis, it is possible to identify numerical and structural abnormalities, which aid in diagnosing syndromes and diseases and in analyzing the evolutionary history of species [1,2]. In veterinary medicine, chromosomal approaches are relevant to genetic improvement and conservation programs [3,4,5]. Among animals, felids are a diverse group found on nearly every continent and comprising approximately 40 species [6]. This group includes species of ecological and biological importance, as well as veterinary significance, particularly the domestic cat, *Felis catus* [7]. From a cytogenetic perspective, felids exhibit highly conserved karyotypes, with most species having 2n = 38 chromosomes, the sole exception being the genus *Leopardus*, which has 2n = 36 [6,8,9]. Despite this apparent karyotypic conservation, obtaining high-quality metaphase chromosomes in felids remains technically challenging.

Over the past century, the karyotypes of various feline species have been described, frequently relying on tissue samples for fibroblast culture [8,9,10,11,12,13,14,15]. Although effective, these approaches are invasive, labor-intensive, and often impractical for wild or endangered species, where ethical and logistical constraints limit sample collection. Peripheral blood represents a minimally invasive and widely accessible alternative source of dividing cells [11,12,16]. However, successful chromosome preparation depends on efficient mitogenic stimulation to induce lymphocyte proliferation. Phytohemagglutinin (PHA) and pokeweed mitogen (PWM) are well-established T-cell mitogens that have been successfully applied in cytogenetic studies of mammals and reptiles [11,17,18]. In felids, several studies have demonstrated that PHA or PWM can produce high-quality metaphase chromosomes suitable for cytogenetic analyses (e.g., [1,8,12,19]). Nevertheless, most published protocols do not provide sufficient methodological detail to facilitate reproducibility across laboratories, and key experimental parameters, including the volume of peripheral blood used to establish cultures, are often omitted. As a result, direct comparisons among available methodologies are challenging.

Given these limitations, there is a need for a standardized, fully described protocol that can be readily reproduced and broadly applied across felid species. In this context, Concanavalin A (ConA) represents a promising alternative mitogen. ConA is a plant lectin isolated from the jack bean (*Canavalia ensiformis*) that functions as a potent T-lymphocyte mitogen. By binding to specific carbohydrate residues on the lymphocyte surface, ConA stimulates quiescent T cells to re-enter the cell cycle and undergo mitosis, making it a valuable reagent for chromosome analysis in peripheral blood cultures and for detecting numerical and structural chromosomal abnormalities [20,21]. Although ConA has previously been applied to feline lymphocyte cultures [16], its use was restricted to the domestic cat, and several key methodological parameters essential for its reproducibility were not reported. Therefore, the objective of the present study was to develop and validate a comprehensive ConA-based protocol that specifies all critical experimental parameters, including the blood volume required for successful culture establishment, and to evaluate its reproducibility across different domestic and wild felid species.

## 2. Experimental Design

### 2.1. Principle of the Protocol

The protocol is based on the capacity of concanavalin A (ConA), a plant lectin isolated from *Canavalia ensiformis,* to induce the proliferation of peripheral blood T lymphocytes in felids. In resting peripheral blood, most lymphocytes remain in the G0 phase of the cell cycle and therefore do not undergo spontaneous mitosis. ConA binds specifically to α-D-mannosyl and α-D-glucosyl carbohydrate residues on glycoproteins of the lymphocyte plasma membrane, triggering intracellular signaling pathways that promote cellular activation, entry into the cell cycle, DNA synthesis, and progression to mitosis [20,21]. Because chromosome preparations require cells arrested in metaphase, efficient mitogenic stimulation is a critical determinant of cytogenetic success. The present protocol exploits the stronger and more reproducible mitogenic activity of ConA to maximize the number of dividing lymphocytes, thereby generating well-spread metaphase chromosomes suitable for conventional karyotyping as well as downstream molecular cytogenetic applications, including chromosome banding, fluorescence in situ hybridization (FISH), and comparative genomic analyses. The optimized culture conditions described here were established to provide robust and reproducible chromosome preparations from small volumes of peripheral blood while minimizing sample collection and handling, making the protocol particularly suitable for domestic and wild felid species.

### 2.2. Workflow Overview

The protocol comprises a sequential workflow designed to obtain high-quality metaphase chromosomes from peripheral blood lymphocytes of felids. Initially, peripheral blood is collected into heparinized tubes and inoculated into supplemented RPMI-1640 culture medium containing fetal bovine serum, antibiotics, L-glutamine, and ConA. During a 70 h incubation period at 37 °C in a humidified atmosphere containing 5% CO_2_, ConA activates quiescent T lymphocytes, stimulating their entry into the cell cycle and promoting cell proliferation. Shortly before the end of the culture period, colchicine is added to arrest dividing cells in the metaphase by disrupting spindle fiber formation, thereby maximizing the recovery of chromosomes suitable for cytogenetic analyses.

**Figure 1:**
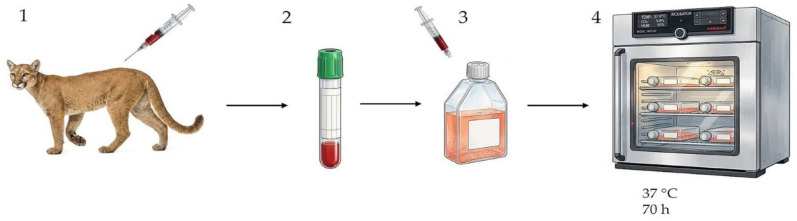
Workflow of felid lymphocyte cell culture. Schematic representation of the main steps involved in establishing peripheral blood lymphocyte cultures, from blood collection and culture initiation to incubation under standardized conditions prior to metaphase chromosome preparation. Illustrations were adapted from the NIAID NIH BIOART Source (bioart.niaid.nih.gov).

**Figure 2:**
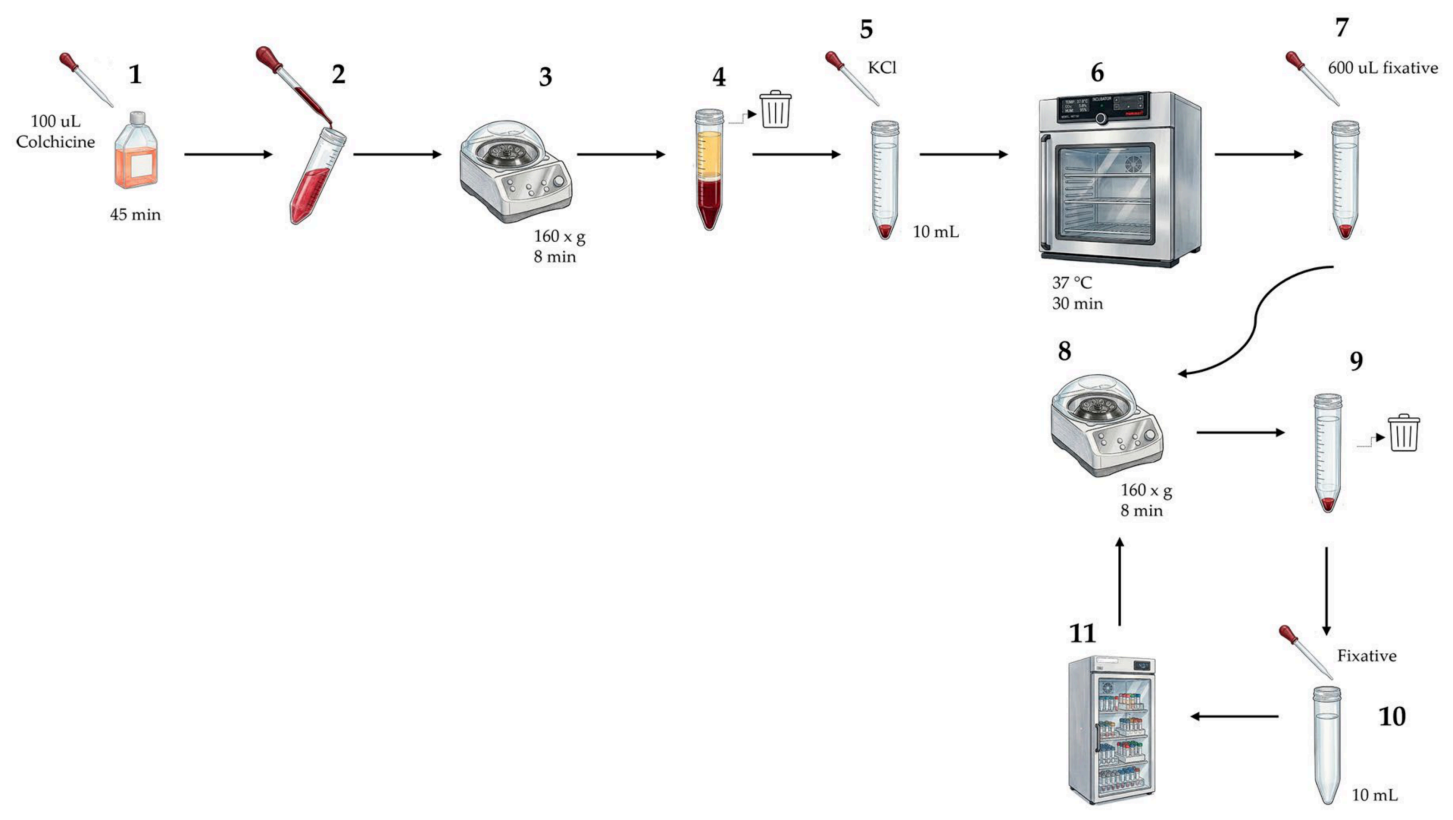
Workflow for metaphase chromosome preparation from felid peripheral blood lymphocyte cultures. Schematic representation of the chromosome preparation procedure, illustrating the sequential stages of colchicine treatment, hypotonic swelling, fixation, and sample storage prior to slide preparation for cytogenetic analysis. Illustrations were adapted from the NIAID NIH BIOART Source (bioart.niaid.nih.gov).

Following mitotic arrest, cultured cells are subjected to hypotonic treatment with pre-warmed potassium chloride to induce controlled cellular swelling and improve chromosome dispersion. Cells are subsequently fixed through repeated washes with freshly prepared methanol–acetic acid (Carnoy’s fixative) to preserve chromosomal morphology and remove cytoplasmic components. The resulting cell suspension is dropped onto preheated microscope slides, allowing chromosomes to spread adequately during air drying before Giemsa staining. The prepared metaphase spreads are then examined under a light microscope for chromosome counting, karyotype construction, and downstream cytogenetic applications, including chromosome banding, fluorescence in situ hybridization (FISH), and comparative chromosome analyses. The overall workflow is illustrated in Figure 1 and Figure 2 and serves as the conceptual framework for the detailed step-by-step protocol presented in the following sections.

### 2.3. Choice of Culture Conditions

Blood cultures provide many advantages compared to fibroblast cultures, such as reduced cost, minimal infrastructural demands, and reduced handling, which therefore lowers the potential risk of contamination. The efficacy of blood cultures relies on the presence of circulating lymphocytes in peripheral blood and the effectiveness of mitogens in promoting cell division. Lymphocytes constitute between 20 and 45% of total leukocytes in domestic cats [22,23,24,25]. Plant lectins constitute some of the most often utilized mitogens, as they are recognized for inducing lymphocyte proliferation by promoting cellular entry into the cell cycle. This process increases the mitotic index and enhances the yield of metaphase spreads for cytogenetic investigation [26]. Previous studies have employed ConA at concentrations ranging from 5 to 25 μg/mL, primarily for immunological assays rather than chromosome preparation [22,23,24,27]. Furthermore, essential experimental parameters, including final mitogen concentration, blood volume, incubation time, and chromosome preparation procedures, have varied considerably or remained incompletely reported, preventing protocol reproducibility. The present study addresses this gap by defining standardized culture conditions centered on a final ConA concentration of 40 μg/mL and integrating all subsequent cytogenetic procedures into a single validated workflow.

The final culture conditions were established following preliminary optimization assays evaluating the relationship between blood volume and ConA concentration, together with incubation time. Different combinations of blood volume and mitogen concentration were tested to identify conditions that maximized metaphase yield while minimizing blood requirements. Increasing both blood volume (>500 μL) and ConA concentration (100 μg/mL) did not improve chromosome preparations and resulted in a poor metaphase yield (<10 metaphases per slide, SD ± 5). This outcome was likely associated with excessive mitogenic stimulation and increased cell density. Conversely, reducing the ConA concentration (8 μg/mL) while increasing the blood volume (500 μL) produced satisfactory metaphase preparations. However, this condition required a larger blood sample, which was considered less desirable, particularly for applications involving small or wild felids. The combination of 300 μL of whole blood with 40 μg/mL ConA consistently produced the highest number of well-spread metaphases while preserving chromosome morphology and minimizing blood collection requirements.

Although the 72 h incubation period is well established for mammalian protocols, our findings demonstrate that a 70 h incubation maintains satisfactory and reproducible results. This shift to a 70 h window allows for protocol optimization by reducing overall processing time.

To evaluate the robustness and reproducibility of the protocol, we validated it using peripheral blood samples from eight representative felids, including the domestic cat (*Felis catus*), the cougar (*Puma concolor*), the jaguar (*Panthera onca*), the lion (*Panthera leo*), the jaguarundi (*Herpailurus yagouaroundi*), the margay (*Leopardus wiedii*), the ocelot *Leopardus pardalis*, and the oncilla *Leopardus tigrinus*. All felids were sampled from zoological institutions or Wildlife Screening and Rehabilitation Centers (CETRAS) in Brazil, except for the domestic cat sample, which was obtained from privately owned animals in the municipality of São Carlos, São Paulo, Brazil. Sample collection was conducted under SISBIO permit No. 101397-1, and all experimental procedures were approved by the Animal Experimentation Ethics Committee of the Federal University of São Carlos (CEUA-UFSCar; Protocol No. 4100161025, approved on 16 October 2025). These species represent both domestic and wild felids, encompassing both small- and large-bodied taxa with diverse ecological characteristics. The protocol was applied using identical culture conditions across all species to assess its reproducibility and broad applicability, demonstrating its suitability as a standardized method for cytogenetic studies throughout the family Felidae.

## 3. Materials, Reagents, Solutions, and Equipment

This section lists the materials, reagents, and solutions used in the protocol, along with guidance for preparing the corresponding working solutions when applicable. Table S1 lists the laboratory equipment required to run this protocol.

### 3.1. Materials

The following materials are required in the study protocol:

- Adjustable micropipettes;
- Aluminum foil;
- Amber glass bottle;
- Biohazard disposal bags;
- Cell culture flasks with filter (25 cm^2^);
- Glass microscope slides (pre-cleaned);
- VACUETTE^®^ 2 mL LH Heparin Tube for Lytic Testing (Cat. Nr. 454237, Greiner Bio-One, Kremsmünster, Austria);
- Immersion oil;
- Microcentrifuge tube racks;
- Plastic Pasteur pipettes;
- Racks for Falcon tubes (15 mL and 50 mL);
- Serological pipettes (5 mL, 10 mL);
- Slide staining rack or Coplin jars;
- Sterile microcentrifuge tubes (1.5 mL);
- Sterile 15 mL Falcon tubes;
- Sterile pipette tips of various volumes;
- Sterile plastic Pasteur pipettes;
- Sterile disposable 1 mL syringes with 21–25-gauge (21–25 G) hypodermic needles.

### 3.2. Reagents

The following reagents are required in the study protocol:

- Acetic acid (glacial, ≥99.7%, Cat. No. 695092, Sigma-Aldrich, St. Louis, MO, USA).
- Fetal Bovine Serum (FBS) Qualified (Cat. No. 26140079, Thermo Fisher Scientific, Waltham, MA, USA).
- Concanavalin A from Canavalia ensiformis Type IV-S (Cat. No. C0412, Sigma-Aldrich, St. Louis, MO, USA).
- Colchicine (Cat. No. C9754, Sigma-Aldrich, St. Louis, MO, USA). *Note:* Handle with appropriate safety precautions, as colchicine is a highly cytotoxic agent.
- Ethanol 70% (Cat. No. EX0281, Merck, Darmstadt, Germany).
- Giemsa stain solution (Cat. No. 10092013, Thermo Fisher Scientific, Waltham, MA, USA).
- Glycerol (C_3_H_8_O_3_) (Cat. No. G7893, Merck, Darmstadt, Germany).
- L-Glutamine (Cat. No. G7513, Sigma-Aldrich, St. Louis, MO, USA).
- Methanol (Cat. No. 322415, Sigma-Aldrich, USA).
- Penicillin-Streptomycin-Amphotericin B (Cat. No. A5955, Sigma-Aldrich, St. Louis, MO, USA).
- Phosphate-Buffered Saline 10× (PBS) (Cat. No. 59331C, Sigma-Aldrich, St. Louis, MO, USA).
- Potassium chloride (KCl) (Cat. No. P9541, Sigma-Aldrich, St. Louis, MO, USA).
- Potassium phosphate (KH_2_PO_4_) (Cat. No. 529568, Merck, Darmstadt, Germany).
- RPMI-1640 Medium with L-Glutamine and HEPES (Cat. No. 22400089, Thermo Fisher Scientific, Waltham, MA, USA).
- Sodium phosphate (NaH_2_PO_4_) (Cat. No. 609773, Merck, Darmstadt, Germany).

### 3.3. Solutions

#### 3.3.1. Fetal Bovine Serum (FBS) Aliquots

Under sterile conditions, place aliquot fetal bovine serum into sterile 50 mL Falcon tubes. Store the aliquots at −20 °C according to the manufacturer’s recommendations. Thaw at room temperature or in a 37 °C water bath right before use. Avoid repeated freeze–thaw cycles.

#### 3.3.2. 1× Phosphate-Buffered Saline (PBS)

Prepare 1× PBS by diluting 10 mL of 10× PBS with 90 mL of sterile Milli-Q water. Sterilize by autoclaving and store in a sterile 50 mL Falcon tube at 4 °C.

#### 3.3.3. Concanavalin A (ConA) Stock Solution (5 mg mL^−1^)

Dissolve 5 mg of concanavalin A in 1 mL of sterile 1× PBS. Sterilize the solution by filtration through a 0.22 µm membrane filter under aseptic conditions. Dispense into 1.5 mL microcentrifuge tubes and store at −20 °C.

#### 3.3.4. Supplemented RPMI Culture Medium (With ConA)

Prepare the sample fresh on the day of collection. For each sample, prepare 5 mL of RPMI medium supplemented with 20% fetal bovine serum (FBS), 1% L-glutamine, and 1% antibiotic solution. Finally, add 40 µL of concanavalin A stock solution to obtain a final concentration of 40 µg/mL. Store at 4 °C in a sterile 15 mL Falcon tube and prewarm to 37 °C before use.

#### 3.3.5. Colchicine Solution (0.0125%)

Dissolve 6.25 mg of colchicine in 50 mL of ultrapure Milli-Q water. Store the solution in an amber glass bottle to protect it from light.

#### 3.3.6. Hypotonic Solution (0.075 M KCl)

Dissolve 5.591 g of potassium chloride (KCl; molecular weight 74.55 g mol^−1^) in approximately 800 mL of distilled water. Adjust the final volume to 1 L with distilled water. Store in a 1 L glass bottle at 4 °C and pre-warm to 37 °C before use.

#### 3.3.7. Carnoy’s Fixative (Methanol–Acetic Acid, 3:1)

Prepare fresh by mixing methanol and glacial acetic acid in a 3:1 ratio (*v*/*v*). Store at −20 °C in a sterile 50 mL glass bottle until use.

#### 3.3.8. Giemsa Stock Solution

Dissolve 1 g of Giemsa powder in 54 mL of glycerol and heat to 60 °C. After cooling, add 84 mL of methanol and filter the solution. Aliquot them into 15 mL Falcon tubes, wrap them in aluminum foil to protect them from light, and keep them at room temperature for up to one year.

#### 3.3.9. Phosphate Buffer (pH 6.8)

Prepare two solutions separately: (A) dissolve 4.0827 g of KH_2_PO_4_ in 500 mL of distilled water; (B) dissolve 3.1888 g of Na_2_HPO_4_ in 500 mL of distilled water. Gradually add solution A to solution B while monitoring the pH until it reaches 6.8. Store it in a sterile 100 mL glass bottle, wrap it in aluminum foil, and keep it at room temperature.

#### 3.3.10. Giemsa Working Solution

Prepare immediately before use by mixing 0.5 mL of Giemsa stock solution with 9.5 mL of phosphate buffer (pH 6.8) in a sterile 20 mL glass beaker.

## 4. Detailed Procedure

### 4.1. Sampling and Culture Establishment

Peripheral blood samples from felines can be obtained by jugular venipuncture or from other accessible peripheral veins, depending on factors such as the animal’s size, temperament, and vascular accessibility (Figure 1, step 1). The attending veterinarian should determine the most appropriate venipuncture site, needle gauge (typically 21–25 G), and sedation or restraint protocol according to the species, body size, clinical condition, temperament, and institutional animal welfare guidelines. Before blood collection, animals should be appropriately restrained or sedated, when necessary, following approved ethical and veterinary sedation protocols. The venipuncture site should be disinfected with 70% ethanol before sample collection. Immediately after collection, samples should be transferred to a heparinized tube and gently mixed for ~1 min to prevent clotting (Figure 1, step 2). When transport to the laboratory is required, samples should be maintained under refrigerated conditions (4–10 °C) until processing.

Before establishing the culture, disinfect the laminar flow cabinet with 70% ethanol and arrange all necessary sterile supplies in the workspace, including culture flasks and sterile syringes. The cabinet must be exposed to UV light for a minimum of 25 min before use to ensure proper surface sterilization. Within the laminar flow cabinet, transfer 300 µL of heparinized peripheral blood straight into a culture flask containing pre-warmed enriched culture medium (Figure 1, step 3). This volume allows for one culture flask; when greater blood volumes are accessible, additional flasks may be prepared to enhance lymphocyte production. Blood must be dispensed gradually using a sterile 1 mL syringe to facilitate gentle mixing with the medium. Then, incubate the culture flasks at 37 °C in 5% CO_2_ for 70 h (Figure 1, step 4). During incubation, cultures should be gently mixed every 12 h to ensure uniform cell growth and nutrient distribution.

### 4.2. Mitotic Chromosomal Preparation

Colchicine (100 µL) is added 45 min before completion of the 70 h incubation, and cultures are maintained at 37 °C to induce metaphase arrest. Following colchicine treatment, transfer the cell suspension to a sterile 15 mL Falcon tube and centrifuge at 160× *g* for 10 min (Figure 2, steps 2 and 3). A buffy coat enriched in leukocytes should be visible at this stage. Cells are then resuspended in 10 mL of pre-warmed hypotonic solution (Solution 7), gently mixed by pipetting, and incubated at 37 °C for 30 min (Figure 2, steps 4–6).

Following hypotonic treatment, add 0.6 mL of cold Carnoy I fixative (Solution 8) to each tube and gently mix by pipetting before centrifugation (Figure 2, step 7). Centrifuge the samples at 160× *g* for 8 min, then discard the supernatant, leaving ~1 mL above the pellet (Figure 2, steps 8 and 9). Resuspend the cells in 10 mL of cold Carnoy I fixative (Solution 8) and incubate at 4 °C for 20 min (Figure 2, steps 10–11).

Centrifuge again at 160× *g* for 8 min and discard the supernatant, leaving ~1 mL above the pellet (Figure 2, step 8). Repeat this washing step twice (Figure 2, steps 8–11). After the final centrifugation, discard the supernatant and resuspend the pellet in ~2 mL of Carnoy I fixative (Solution 8), adjusting the volume according to cell density. Store the resulting cell suspension at −20 °C. For chromosome preparations, drop 40 µL of the cell suspension onto different regions of a clean glass microscope slide preheated to ~50 °C, using a micropipette. Allow the preparations to air dry, then stain the slides with Giemsa working solution (Solution 11) for 8 min. Rinse the slides in running water and allow them to air-dry before analysis.

## 5. Expected Results

**Figure 3:**
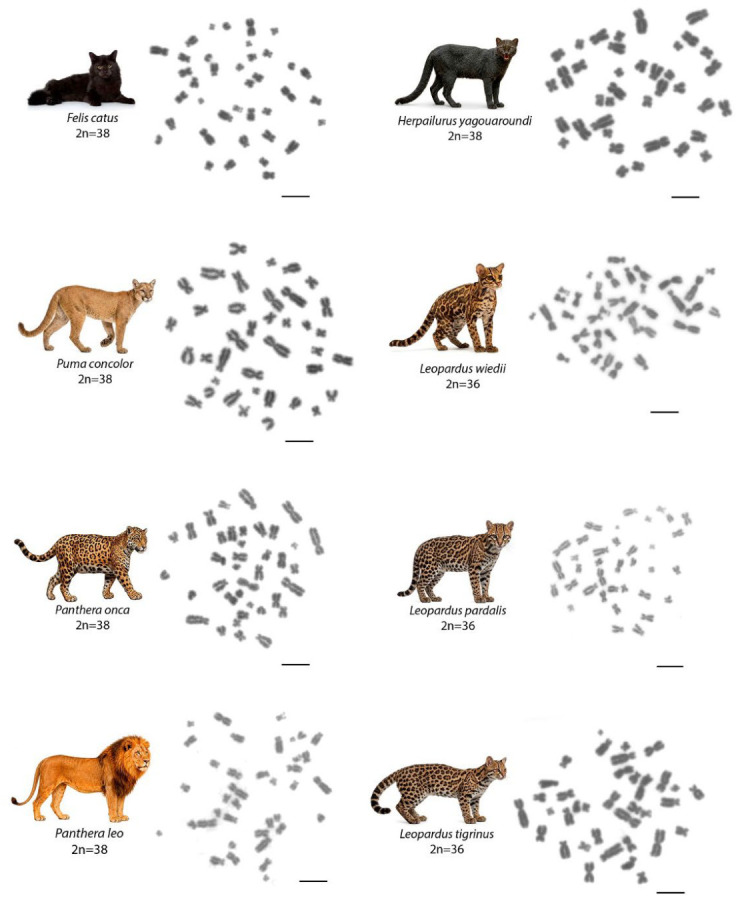
Representative metaphase chromosome spreads from eight felid species generated using the optimized protocol described in this study. Scale bars = 10 µm. Illustrations were adapted from the NIAID NIH BIOART Source (bioart.niaid.nih.gov).

**Figure 4:**
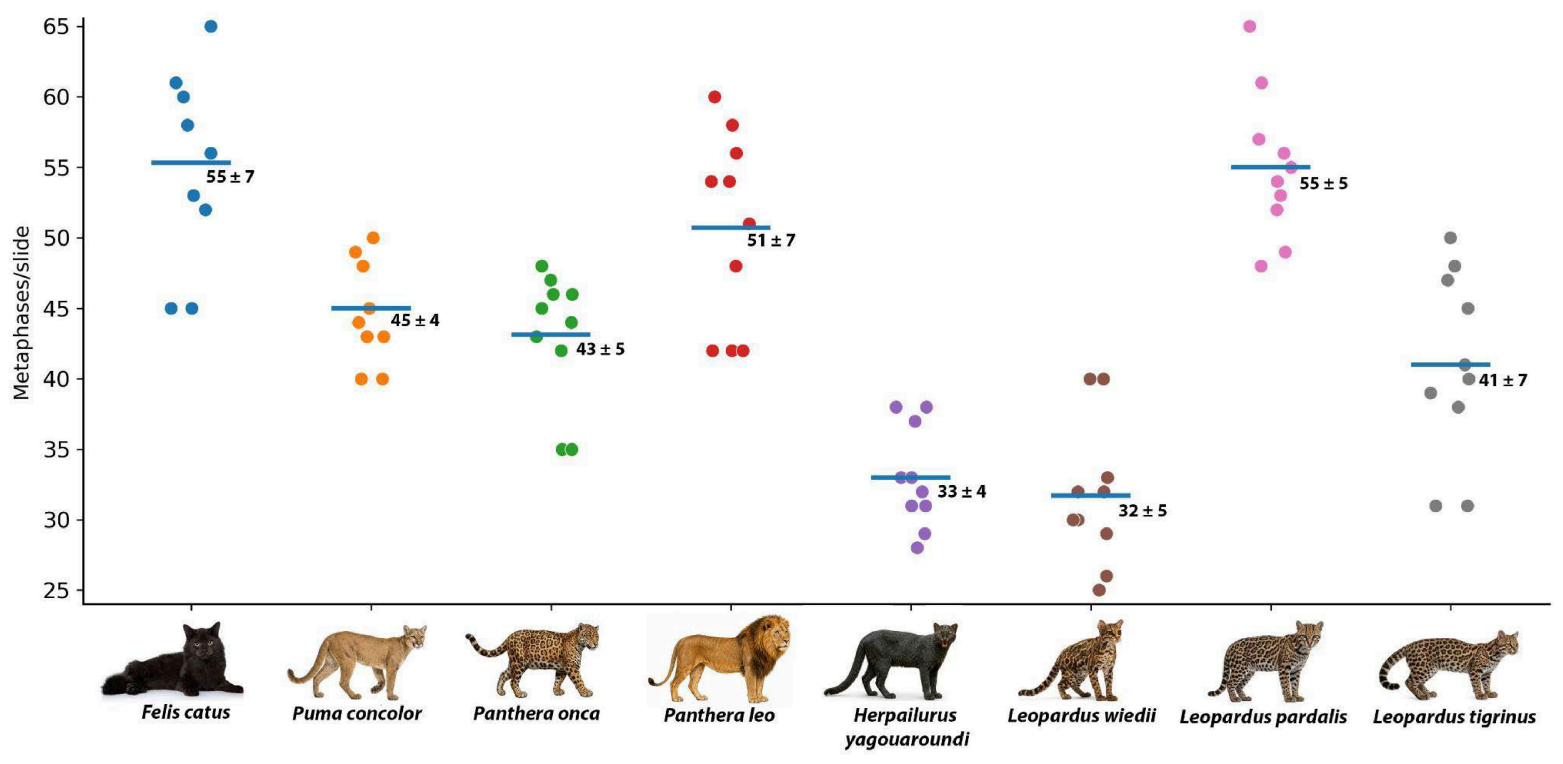
Reproducibility of metaphase yield across eight felid species cultured using the ConA-based lymphocyte protocol described here. Each dot represents the number of analyzable metaphase spreads obtained from a single independent slide (n = 10 slides per chromosome preparation). Horizontal blue bars indicate the mean number of metaphases per slide, and the corresponding values (mean ± SD) are shown beside each bar. The individual metaphase counts used to generate this figure are provided in Table S2. Representative photographs of each species are shown below the *x*-axis for illustrative purposes and are not presented on scale. Illustrations were adapted from the NIAID NIH BIOART Source (bioart.niaid.nih.gov).

The findings demonstrate that ConA is an effective mitogenic agent for felid lymphocyte cultures. Using the standardized protocol described here, high-quality chromosomal preparations can be obtained from both small felids, such as the domestic cat (*Felis catus*) and the ocelot (*Leopardus pardalis*), as well as larger felids, such as the cougar (*Puma concolor*) or the lion (*Panthera leo*). Across all felid species analyzed, the protocol consistently yielded a substantial number of well-spread, analyzable metaphase chromosomes suitable for conventional cytogenetic analyses (Figure 3 and Figures S1–S8). Ten independently prepared microscope slides obtained from the same blood culture for each species (technical replicates) were evaluated, and the mean number of metaphases per slide ranged from 32 ± 5 in *Leopardus wiedii* to 55 ± 7 in *Felis catus*. Observed ranges varied from 25–40 metaphases per slide in *L. wiedii* to 45–65 in *F. catus* and 48–65 in *L. pardalis* (Figure 4 and Table S2). Overall, the protocol consistently produced approximately 44 high-quality metaphases per slide across species, demonstrating high reproducibility and robustness for both domestic and wild felids and providing sufficient material for routine karyotyping and downstream molecular cytogenetic applications. These results were obtained under optimized pre-analytical conditions, including blood collection with minimal restraint-induced stress, absence of medications known to interfere with lymphocyte proliferation, storage of heparinized blood at 4 °C, and initiation of cell cultures that occurred within 2 h after collection. In contrast, two additional samples representing two species (*Felis catus* and *Leopardus pardalis*) that were collected or processed under non-optimal conditions, including pharmacological treatment, increased physiological stress during restraint, or prolonged storage (>12 h) before culture initiation, yielded markedly fewer metaphases (mean = 4.5, SD = 1.87, median = 4.5). Although these samples were not included in the formal quantitative analysis, they provide practical evidence that pre-analytical variables can profoundly affect culture success. Therefore, adherence to appropriate sample collection, handling, and processing procedures is critical for maximizing metaphase yield, often exerting a greater influence on culture performance than the culture conditions themselves.

## 6. Discussion

The protocol presented here provides a standardized and reproducible approach for obtaining high-quality metaphase chromosomes from peripheral blood lymphocytes of felids using concanavalin A (ConA) as the mitogenic stimulus. The innovation of this study is the development of a standardized cytogenetic workflow that defines the critical experimental parameters required for reproducible chromosome preparation in felids. For example, Ghotage et al. [16] demonstrated that ConA is more effective in comparison to other lectins in producing mitoses in peripheral blood cultures for cytogenetic applications in domestic cats. In that study, 500 µL of whole blood was cultured in 7.5 mL of supplemented medium with 80 µL of ConA (concentration unspecified), yielding an increased number of metaphase spreads suitable for karyotypic analysis. By contrast, our protocol details and establishes reproducible culture conditions that consistently generate high-quality metaphase chromosomes from both domestic and wild felids.

To directly compare mitogenic performance, we conducted parallel lymphocyte cultures using peripheral blood from domestic cats under identical experimental conditions, replacing ConA with the conventional 2% PHA. Comparative assays were performed using blood volumes ranging from 300 to 500 µL. Across these comparative assays, cultures stimulated with the conventional 2% PHA consistently yielded poor results, with a mean of only 8 ± 5 analyzable metaphases per slide. In addition to the marked reduction in metaphase yield, PHA-stimulated cultures generated chromosome spreads of inferior quality, with poorer chromosome morphology and suboptimal spreading, frequently compromising cytogenetic analyses. Although we cannot exclude the possibility that substantially larger blood volumes might improve PHA-induced mitotic yields, such an approach would markedly increase sample requirements and would be impractical for most cytogenetic applications involving felids, particularly small-bodied, wild, or endangered species where blood collection is inherently limited.

The successful application of this protocol in distinct species (Figure 3 and Figures S1–S8) demonstrates its robustness across phylogenetically diverse representatives of the family Felidae. Despite differences in body size, ecology, and management conditions among these species, identical culture conditions consistently produced high-quality chromosome preparations suitable for conventional karyotyping and molecular cytogenetic analyses. Also, compared with conventional fibroblast cultures, this method requires only a small volume of peripheral blood, eliminating the need for invasive tissue biopsies and prolonged cell culture establishment. These characteristics make the protocol particularly advantageous for veterinary practice, wildlife management, and conservation programs involving threatened species, in which sample collection is often limited by ethical and logistical constraints.

Although the protocol substantially improves chromosome preparation from peripheral blood, some limitations should be considered. As with any lymphocyte culture system, mitotic responses may vary among individuals depending on biological factors such as age, health status, physiological condition, and immune status, which may influence the final metaphase yield. In addition, although the protocol defined 300 µL as the minimum effective volume, sampling from endangered or clinically compromised animals should always follow veterinary recommendations and ethical guidelines. Importantly, we found that blood samples stored at 4 °C for up to 12 h before culture establishment still yielded satisfactory chromosome preparations, albeit not as effectively as freshly collected blood, indicating that the protocol is sufficiently robust for situations in which immediate laboratory processing is not possible. This is particularly advantageous for wildlife conservation programs, where animals are often sampled in remote locations, and transportation to specialized cytogenetic laboratories may require several hours or days. By including eight felid species representing distinct taxonomic groups, we evaluated the robustness and broad applicability of the protocol across a wide phylogenetic range.

Beyond routine karyotyping, the availability of standardized, high-quality metaphase chromosome preparations substantially broadens the range of cytogenetic and genomic applications that can be performed in felids. The protocol is readily compatible with molecular cytogenetic techniques such as fluorescence in situ hybridization (FISH), comparative genomic hybridization (CGH), chromosome painting, and high-resolution physical gene mapping. These approaches provide powerful tools for investigating chromosome evolution, identifying structural rearrangements, and validating genome assemblies generated through long-read sequencing technologies. As increasingly complete chromosome-level genomes become available for felids, robust metaphase preparations will remain essential for integrating sequence-based and cytogenetic information.

## 7. Conclusions

Together, these findings establish a standardized and reproducible workflow for peripheral blood chromosome preparation in felids. Although ConA has previously been recognized as a lymphocyte mitogen, this study defines the culture conditions required for its reliable application in cytogenetics and validates the protocol across multiple felid species. Beyond routine chromosome analysis, this standardized protocol establishes a foundation for modern cytogenomic research in felids. Its compatibility with molecular cytogenetic approaches, including FISH, chromosome painting, CGH, and physical gene mapping, together with its potential application in genome assembly validation and comparative genomics, makes it a versatile resource for evolutionary biology, veterinary genetics, and conservation genomics.

## Data Availability

All data supporting the findings of this study are included in the article/Supplementary Materials. Further inquiries can be directed to the corresponding authors.

## Supplementary Materials

The following supporting information can be downloaded at https://www.mdpi.com/article/10.3390/ijms27156662/s1.

## Abbreviations

The following abbreviations are used in this manuscript:

ConA	Concanavalin A
PHA	Phytohemagglutinin
PKW	Pokeweed mitogen
