# Supplementary material for: Concanavalin A Protocol for Felid Lymphocyte Culture and Chromosome Preparation
Source: Int J Mol Sci. 2026 Jul 26;27(15):6662. doi: 10.3390/ijms27156662 (PMC13466765; doi:10.3390/ijms27156662)

Table S1. List of laboratory equipment required to run this protocol

Equipment	Purpose
Analytical balance (high precision)	Accurate weighing of reagents and chemicals
Autoclave	Sterilization of media, solutions, and laboratory materials
Centrifuge	Cell harvesting and washing steps
Compound light microscope (100× oil immersion objective)	Evaluation of metaphase chromosome preparations
CO_2 incubator for cell culture	Incubation of lymphocyte cultures under controlled temperature and CO_2 conditions
Freezer (-20 °C)	Storage of reagents and prepared solutions
Laminar flow biosafety cabinet with UV light	Aseptic handling of biological samples and cell cultures
Laboratory refrigerator (+4 °C)	Storage of blood samples, reagents, and culture components
Milli-Q ultrapure water purification system	Production of ultrapure water for reagent preparation
Water bath (37 °C)	Warming reagents and maintaining solutions at physiological temperature

Table S2. Validation of the standardized Concanavalin A (ConA)-based lymphocyte culture and chromosome preparation protocol established in this study. Ten independent slides were evaluated per species (Slides 1–10), and the number of well-spread metaphases suitable for cytogenetic analysis was recorded for each slide. The last column presents the mean number of metaphases per slide ± standard deviation (SD).

**Species**	Slide_1	Slide_2	Slide_3	Slide_4	Slide_5	Slide_6	Slide_7	Slide_8	Slide_9	Slide_10	Mean ± SD
Felis catus	45	65	61	56	60	58	52	58	53	45	55 ± 7
Puma concolor	40	50	48	43	48	45	49	43	44	40	45 ± 4
Panthera onca	35	48	47	46	44	42	46	45	43	35	43 ± 5
Panthera leo	42	60	56	42	48	51	54	58	54	42	51 ± 7
Herpailurus yagouaroundi	28	38	37	33	31	31	33	32	38	29	33 ± 4
Leopardus wiedii	25	40	32	32	30	30	29	26	33	40	32 ± 5
Leopardus pardalis	48	65	54	53	49	61	57	52	56	55	55 ± 5
Leopardus tigrinus	31	50	45	47	48	39	38	40	41	31	41 ± 7

Figure S1. Representative metaphase chromosome spreads obtained from peripheral blood lymphocyte cultures of *Felis catus* (2n = 38) using the standardized ConA-based protocol. Eight representative metaphase plates are shown, illustrating the high quality and reproducibility of chromosome preparations. Scale bar = 10 μm.

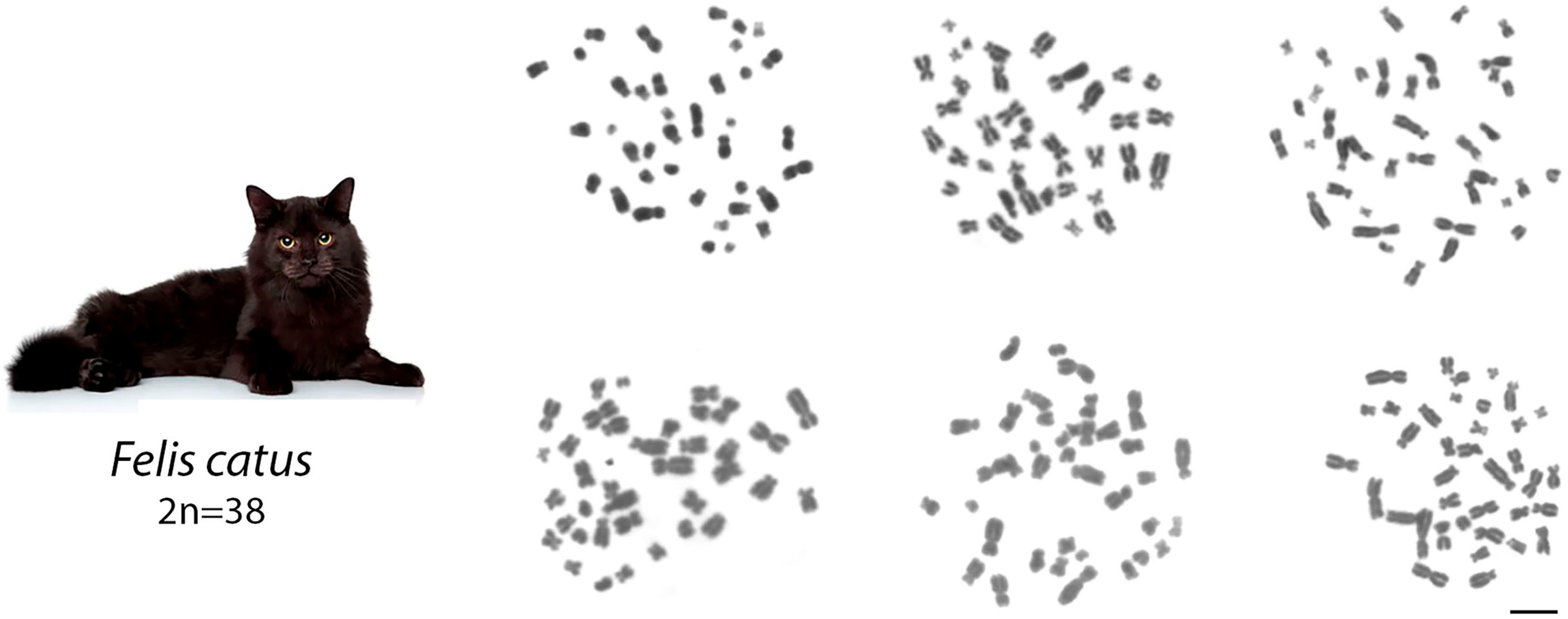

Figure S2. Representative metaphase chromosome spreads obtained from peripheral blood lymphocyte cultures of *Puma concolor* (2n = 38) using the standardized ConA-based protocol. Eight representative metaphase plates are shown, illustrating the high quality and reproducibility of chromosome preparations. Scale bar = 10 μm.

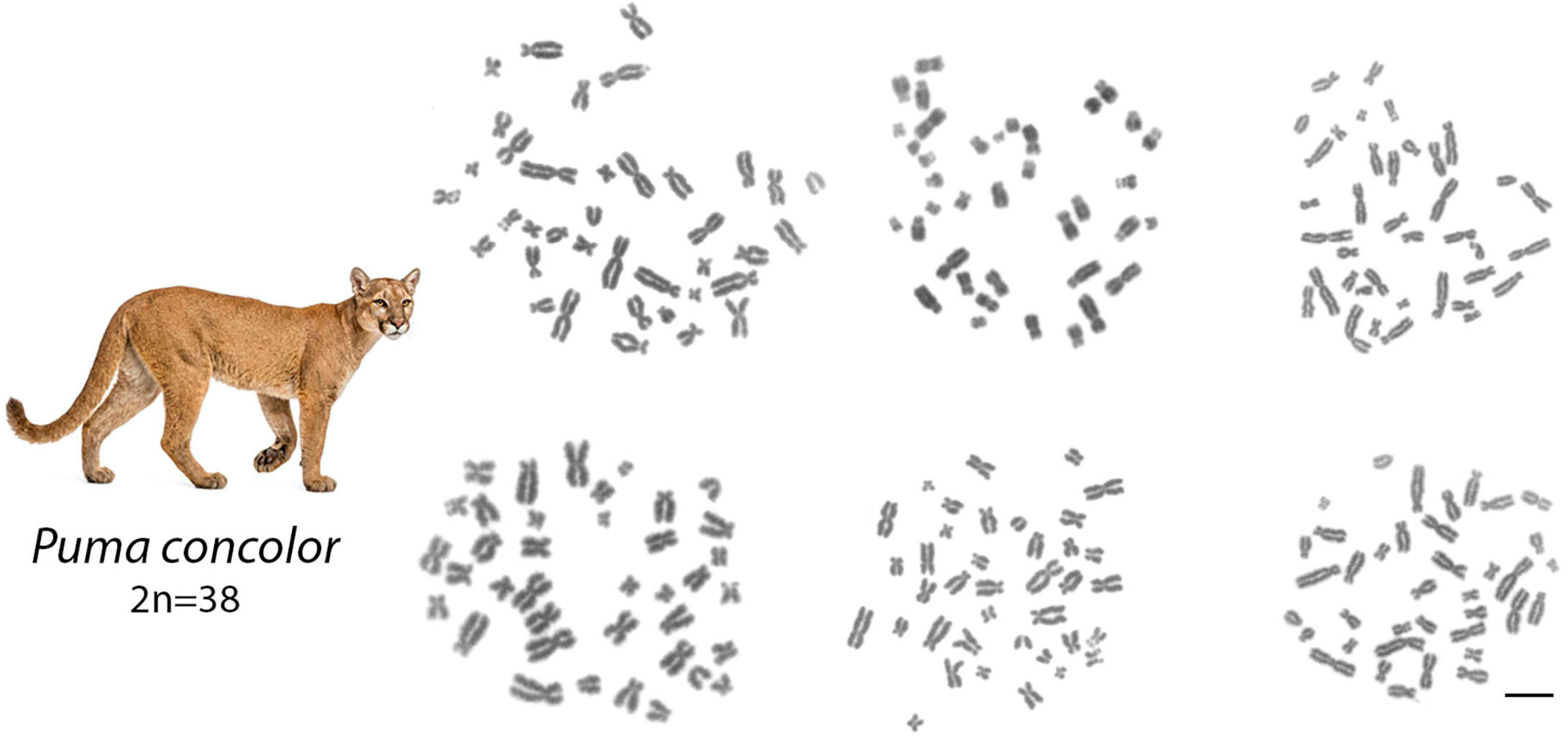

Figure S3. Representative metaphase chromosome spreads obtained from peripheral blood lymphocyte cultures of *Panthera onca* (2n = 38) using the standardized ConA-based protocol. Eight representative metaphase plates are shown, illustrating the high quality and reproducibility of chromosome preparations. Scale bar = 10 μm.

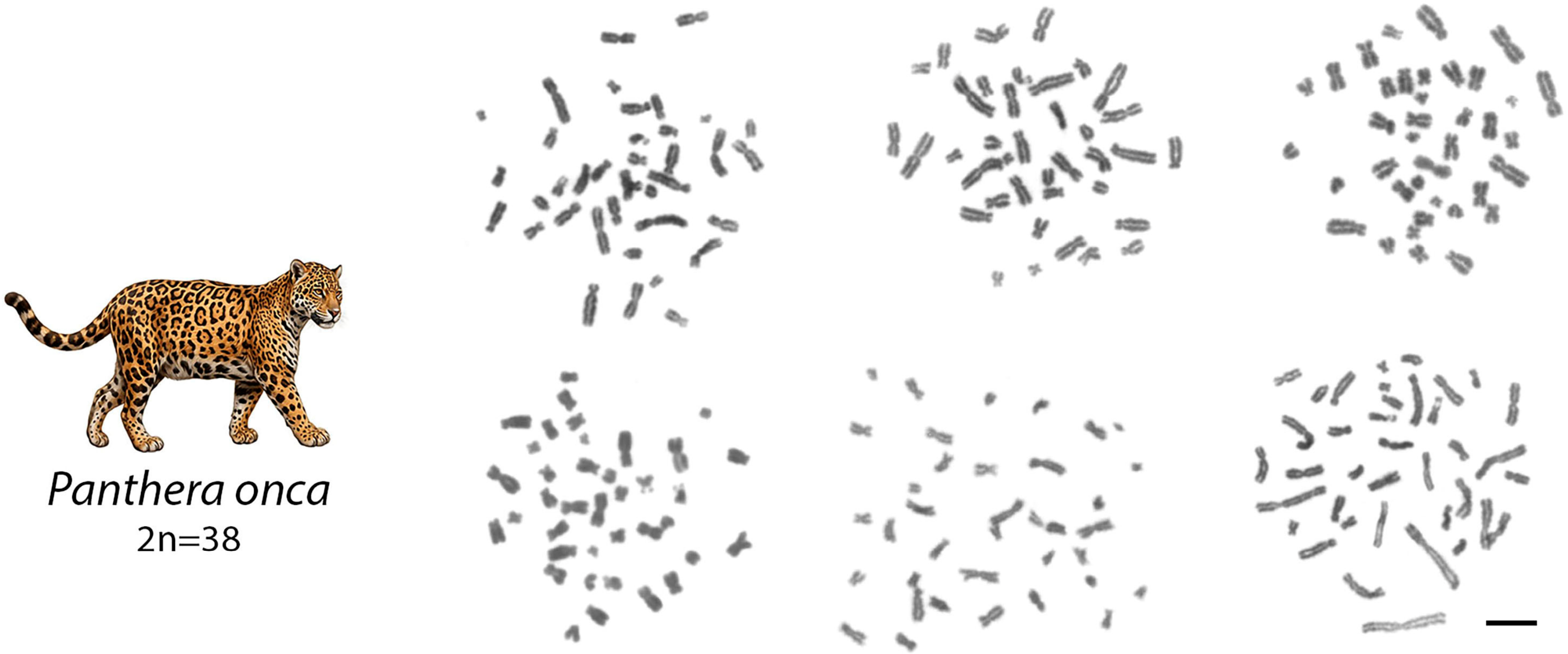

Figure S4. Representative metaphase chromosome spreads obtained from peripheral blood lymphocyte cultures of *Panthera leo* (2n = 38) using the standardized ConA-based protocol. Eight representative metaphase plates are shown, illustrating the high quality and reproducibility of chromosome preparations. Scale bar = 10 μm.

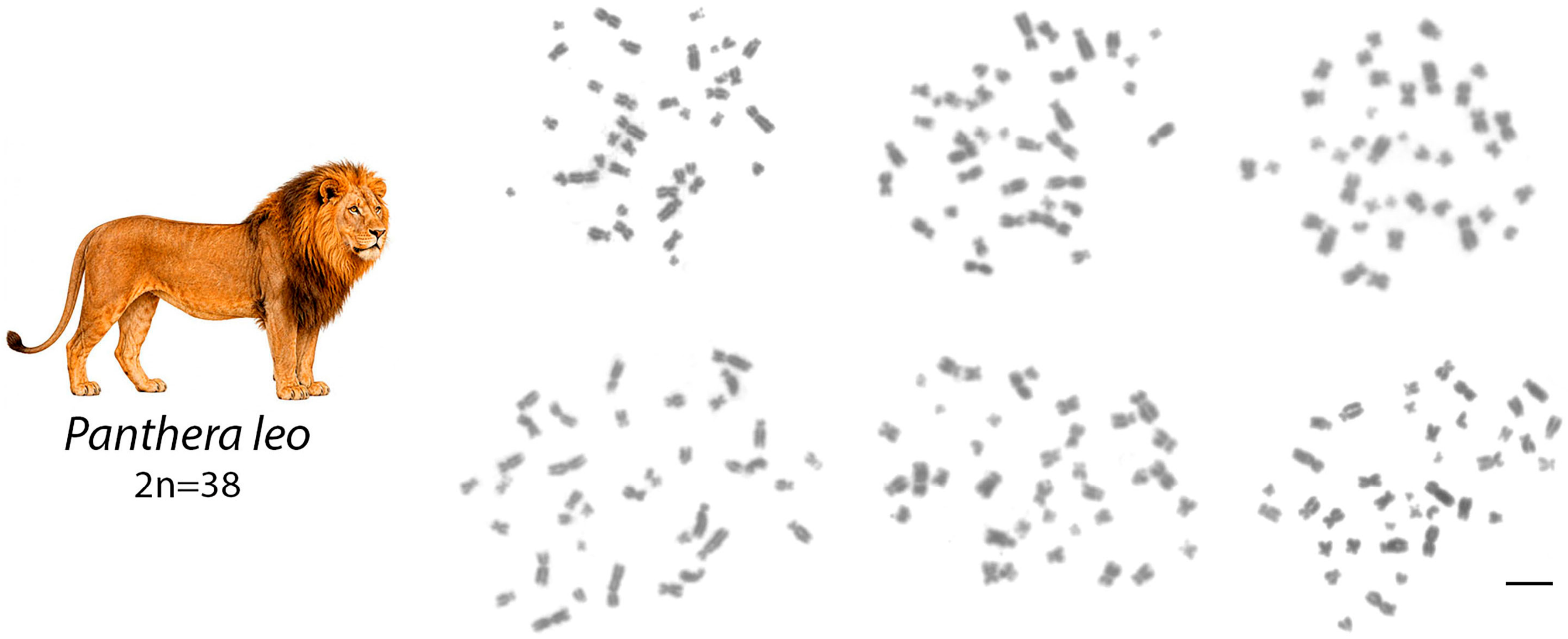

Figure S5. Representative metaphase chromosome spreads obtained from peripheral blood lymphocyte cultures of *Herpailurus yagouaroundi* (2n = 38) using the standardized ConA-based protocol. Eight representative metaphase plates are shown, illustrating the high quality and reproducibility of chromosome preparations. Scale bar = 20 μm.

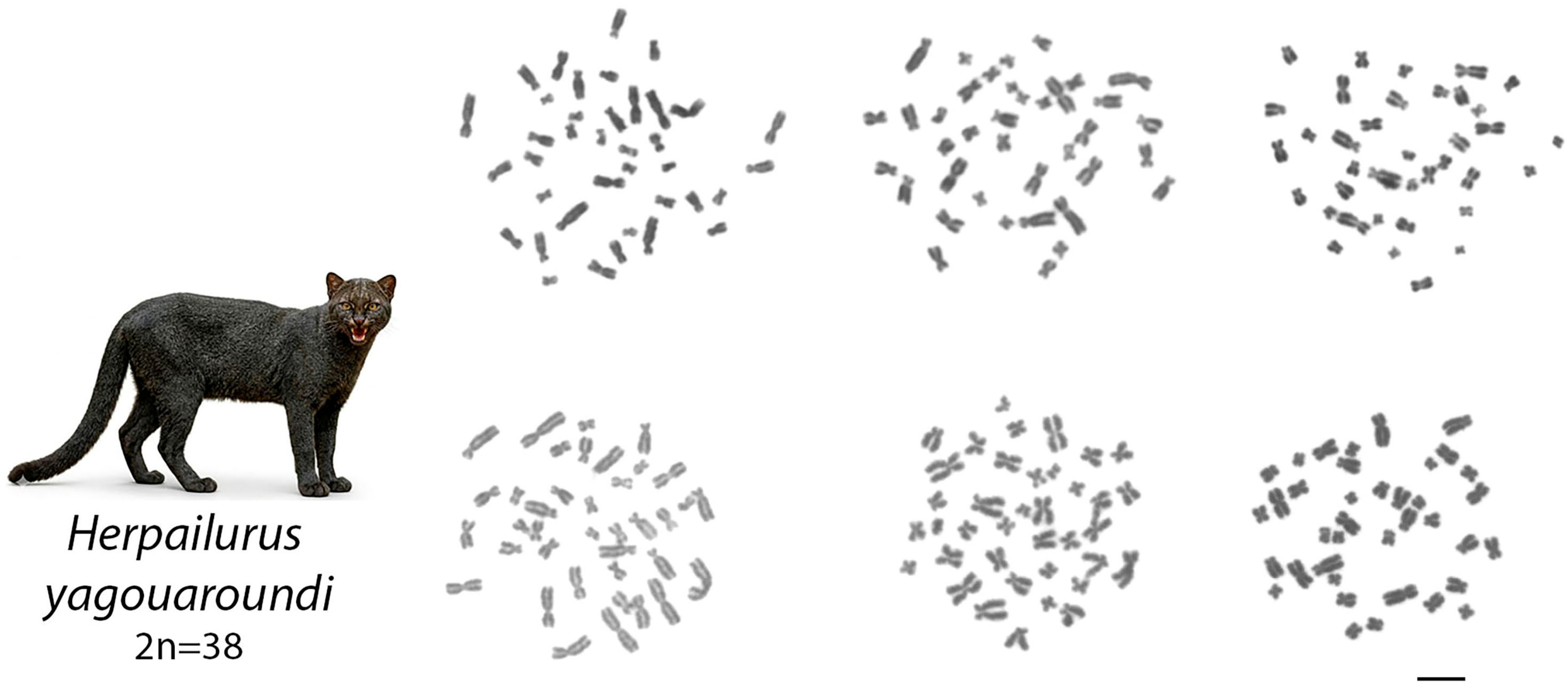

Figure S6. Representative metaphase chromosome spreads obtained from peripheral blood lymphocyte cultures of *Leopardus wiedii* (2n = 36) using the standardized ConA-based protocol. Eight representative metaphase plates are shown, illustrating the high quality and reproducibility of chromosome preparations. Scale bar = 20 µm.

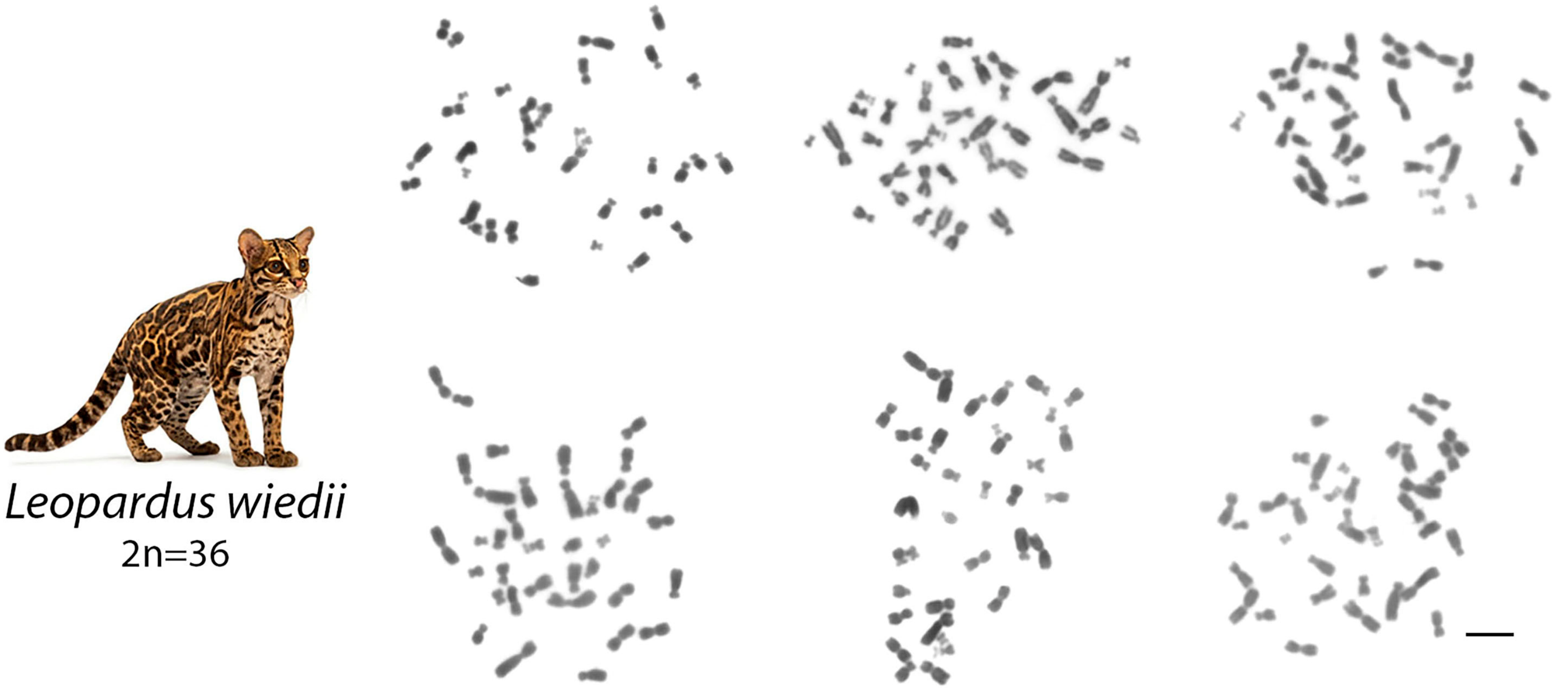

Figure S7. Representative metaphase chromosome spreads obtained from peripheral blood lymphocyte cultures of *Leopardus pardalis* (2n = 36) using the standardized ConA-based protocol. Eight representative metaphase plates are shown, illustrating the high quality and reproducibility of chromosome preparations. Scale bar = 10 µm.

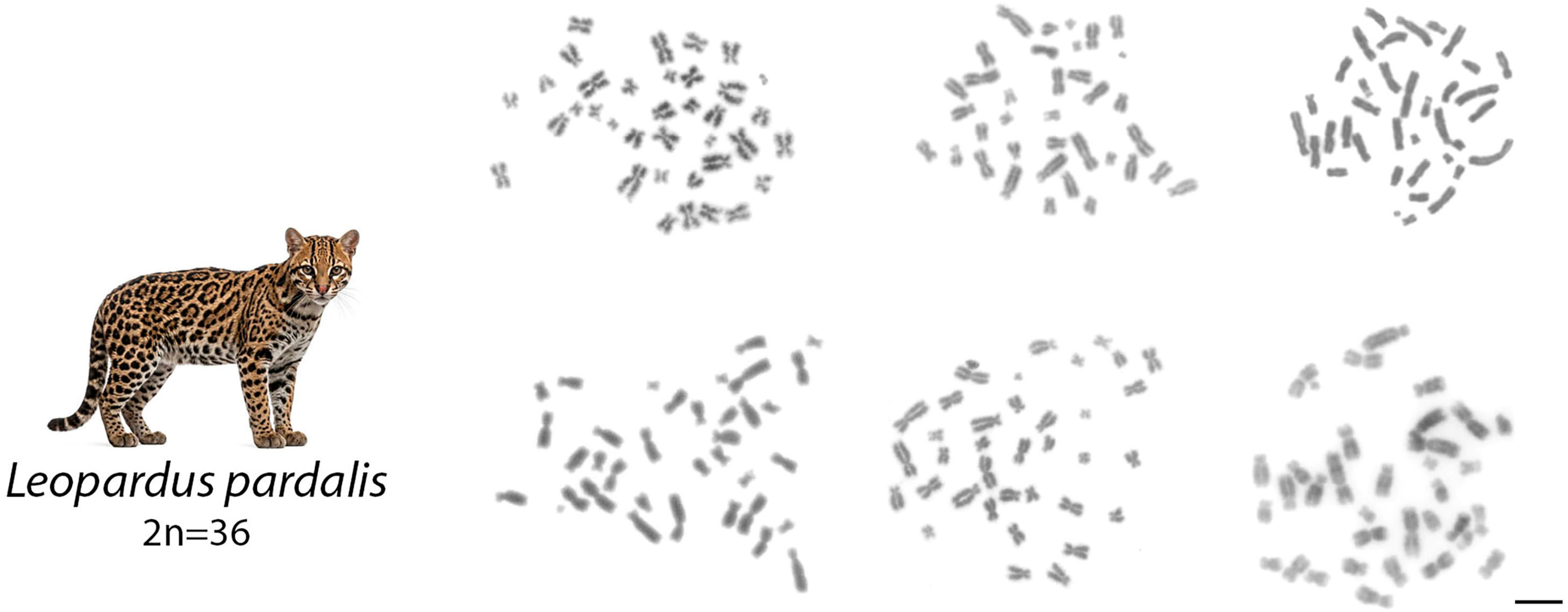

Figure S8. Representative metaphase chromosome spreads obtained from peripheral blood lymphocyte cultures of *Leopardus tigrinus* (2n = 36) using the standardized ConA-based protocol. Eight representative metaphase plates are shown, illustrating the high quality and reproducibility of chromosome preparations. Scale bar = 10 µm.

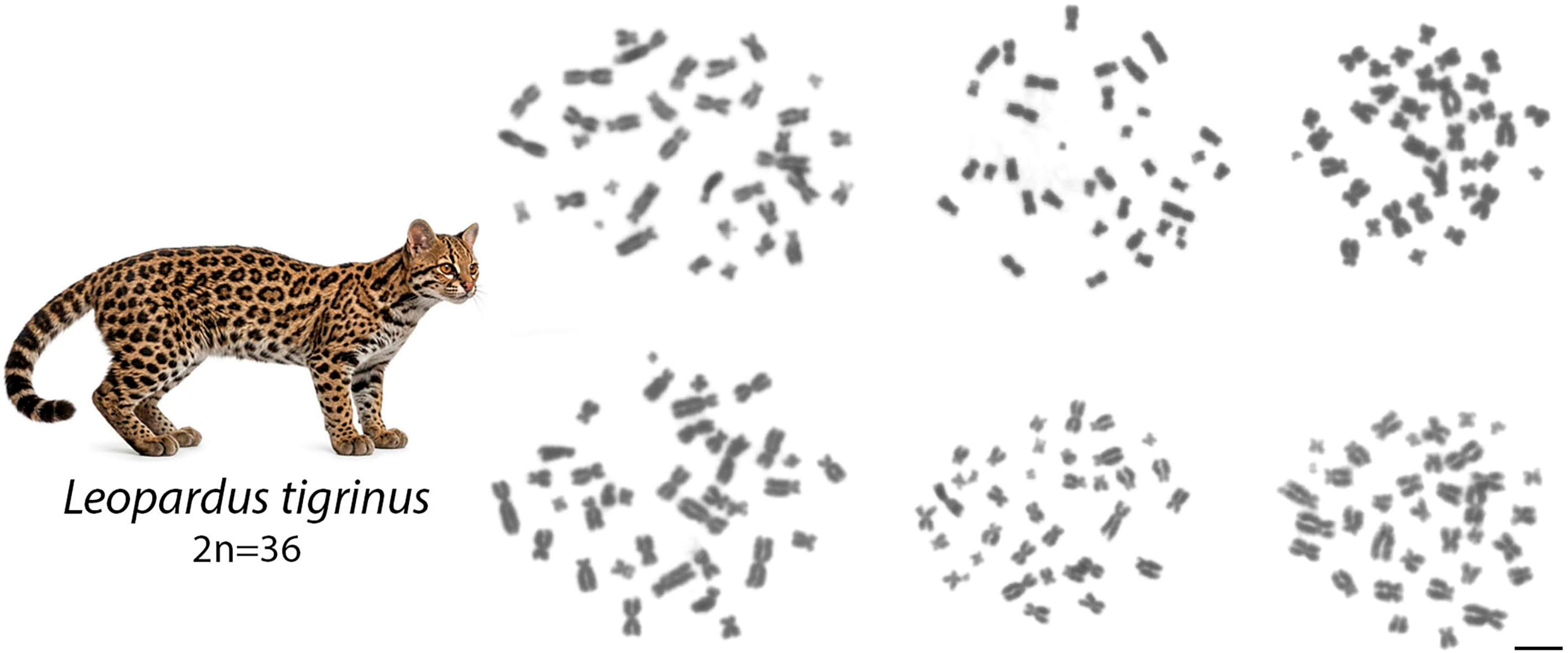

Supplement: Supplementary file 1 [file ijms-27-06662-s001.zip › ijms-4445280-supplementary.pdf]
